# Abrine targets ERK to suppress EMT and lung metastasis model via MAPKs and Nrf2/Keap-1/HO-1 signaling

**DOI:** 10.3389/fimmu.2026.1789110

**Published:** 2026-03-27

**Authors:** Yufang Shen, Linyu Xiao, Lina Liu, Lianting Liao, Xianmin Zou, Pengfei Shen, Jinlong Zhao, Renyikun Yuan, Hongwei Gao, Chun Yao

**Affiliations:** 1 Guangxi University of Chinese Medicine, Traditional Chinese Medicine Zhuang Yao Drug Innovation Drug Engineering Research Center, Nanning, China; 2 College of Pharmacy, Guangxi University of Chinese Medicine, Nanning, China; 3 College of Basic Medicine, Guangxi University of Chinese Medicine, Nanning, China; 4 Guangxi University of Chinese Medicine, Engineering Research Center in Ministry of Education for Innovative Drugs of Traditional Chinese Medicine and Zhuang Yao Medicine, Nanning, China

**Keywords:** Abrine, epithelial-mesenchymal transition (EMT), lung metastasis model, MAPKS pathway, Nrf2/Keap-1/HO-1 pathway

## Abstract

Abrine, an indole alkaloid, was evaluated for its anti-metastatic activity and mechanism in non-small cell lung cancer (NSCLC) models. Human A549 and H1975 cells were exposed to Abrine, showing no cytotoxicity but marked suppression of TGF-β1-induced migration and invasion by scratch and Transwell assays. Mechanistically, Abrine reversed epithelial-mesenchymal transition (EMT) by upregulating E-cadherin and reducing N-cadherin, vimentin, Snail and Slug, findings corroborated by immunofluorescence. Abrine also diminished phosphorylation of ERK, JNK and p38 while modulating the Nrf2/Keap-1/HO-1 axis. Target engagement was supported by molecular docking, CETSA, DARTS, and MST indicating specific binding and thermal stabilization of ERK. *In vivo*, Abrine (20 or 40 mg/kg) mitigated lung metastasis in a B16-F10 intravenous model, improving body weight, decreasing lung metastatic burden, lung wet weight and lung index, and reducing Ki67 expression in lung tissue. Systemic effects included lowered circulating IL-17, IL-10, TNF-α and IFN-γ, and absence of overt histopathological toxicity in major organs. Combination with the ERK1/2 inhibitor SCH772984 further enhanced efficacy. Collectively, Abrine exerts potent anti-metastatic effects by directly targeting ERK to inhibit MAPK signaling, reversing EMT and engaging the Nrf2/Keap-1/HO-1 pathway, highlighting Abrine alone or with ERK inhibition as a promising therapeutic strategy against lung metastasis model.

## 1 Introduction

Lung cancer remains one of the most prevalent and lethal malignancies worldwide. In 2022, an estimated 2.48 million new cases were diagnosed globally, accounting for ~12.4% of all cancers (1, 2). Etiologic drivers include tobacco exposure, air pollution, and genetic susceptibility, while pathogenesis involves carcinogen-induced mutations, chronic inflammation, and remodeling of the tumor microenvironment (TME). Histologically, lung cancer comprises small-cell lung cancer (SCLC) and non-small-cell lung cancer (NSCLC), the latter representing ~80-85% of cases and a principal cause of cancer-related deaths. Metastasis is the major determinant of poor prognosis (3, 4). A critical initiating step is epithelial-mesenchymal transition (EMT), through which tumor cells lose cell-cell adhesion and acquire invasive, migratory phenotypes, facilitating intravasation, dissemination, and colonization at distant sites (5, 6). Hallmarks include loss of the epithelial marker E-cadherin and gain of mesenchymal markers such as N-cadherin and Vimentin (7). EMT is orchestrated by multiple signaling networks, notably the mitogen-activated protein kinase (MAPK) cascade and the Nrf2/Keap-1/HO-1 axis. Consequently, targeting EMT regulators is an attractive strategy to prevent or treat metastatic disease (8).

Despite advances in surgery, radiotherapy, chemotherapy, and targeted therapies, overall survival for NSCLC remains unsatisfactory (9). Many patients with advanced stages or are ineligible for aggressive modalities, and therapy-related toxicities are common (10). Tyrosine kinase inhibitors (TKIs) directed at driver mutations (e.g., EGFR) benefit selected patients, but only a minority harbor actionable mutations, resistance inevitably emerges and clinically significant adverse effects often limit sustained use (11). These limitations underscore the need for safer, mechanism-based therapies that restrain metastatic progression and improve quality of life.

**Figure 1:**
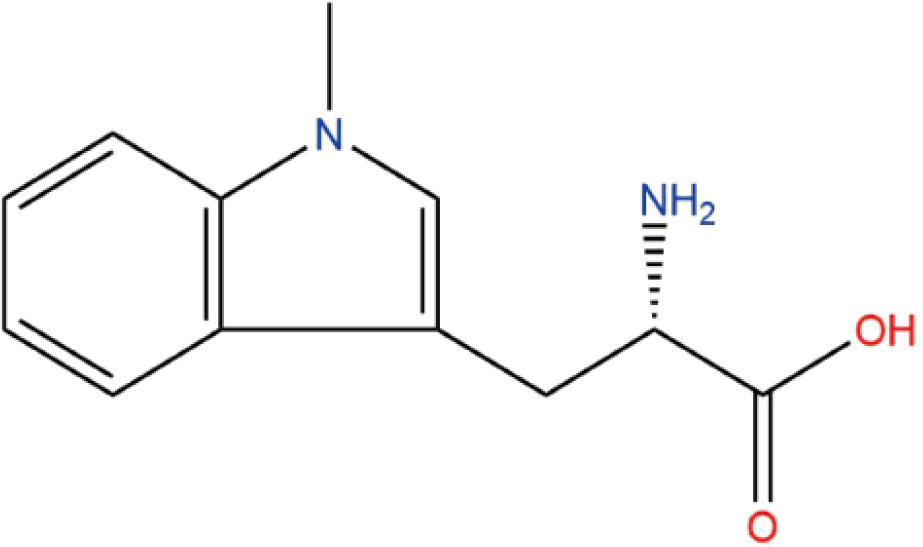
The structure of Abrine.

Abrine is a principal indole alkaloid and a putative bioactive constituent isolated from *Abrus cantoniensis* (Leguminosae) and *Abrus precatorius* Linn, with reported anti-tumor, antioxidant, antibacterial, antiviral, and immunoregulatory activities (12). Such active substances derived from traditional Chinese medicines are precisely one of the important research and development carriers for innovative drugs integrating traditional Chinese medicine and Western medicine (13). In our previous study, Abrine alleviated hepatocellular carcinoma by inhibiting IDO-driven tryptophan catabolism and immune suppression (14), however, its precise mechanisms of action against lung cancer remain to be clarified. In this study, we found that Abrine was non-cytotoxic yet markedly suppressed TGF-β1-induced migration/invasion and reversed EMT related protein expression, inhibited MAPK phosphorylation (ERK/JNK/p38), and modulated the Nrf2/Keap-1/HO-1 axis; In addition, molecular docking, CETSA, DARTS and MST assays confirmed that Abrine direct interaction with ERK. In B16-F10 lung-metastasis mice, Abrine reduced metastatic burden, lung wet weight, improved body weight, and lung pathology, lowered IL-17/IL-10/TNF-α/IFN-γ levels, Inhibit the expression of the tumor proliferation marker Ki67 and showed no overt organ toxicity. Collectively, these findings indicate that Abrine as a candidate for anti-metastatic therapy in lung cancer. As shown in **Figure 1A**, Abrine a natural alkaloid compound-was structurally characterized.

## 2 Materials

### 2.1 Cell lines and culture

The human A549 and H1975 cell lines used in the experiment were purchased from the Shanghai Cell Bank of the Chinese Academy of Sciences. Both cells were cultured in RPMI-1640 complete medium containing 10% FBS and 1% PS (100 U/mL penicillin, 100 μg/mL streptomycin). The murine B16-F10 melanoma cells were obtained from the Cell Bank of the Chinese Academy of Sciences. The cells were cultured in DMEM medium containing 10% FBS and 1% PS, incubated in a 37 °C, 5% CO_2_ incubator. In terms of cell passage, regular subculture was performed according to the cell growth status to maintain the logarithmic growth state. This was usually done every 2–3 days, depending on the cell adhesion and proliferation rate. Cells were split at approximately 70-80% confluence using typsin-EDTA, typically at a 1:3 ratio. Only cells within a limited passage range (<20 passages after thawing) were used for experiments to minimize phenotypic drift. All cell experiments were conducted when the cells were in a good state and in the logarithmic growth phase.

### 2.2 Reagents and instruments

Abrine (purchased from Chengdu Pui Fa Technology Development Co., Ltd., purity: ≥98%); TGF-β1 (#HY-P70543), Sch772984 (#HY-50846), and Gefitinib (#ZD1839) purchased from MedChemExpress (MCE Company, Shanghai, China); RPMI-1640 and FBS purchased from the United States Thermo Fisher Scientific (Gibco) Company; β-actin (#BF8004, Affinity), E-cadherin (#14472S, CST, 1:1000), N-cadherin (#113116S, CST, 1:1000), Vimentin (#5741S, CST, 1:1000), Slug (#9585S, CST, 1:1000), Snail (#3879S, CST, 1:1000), Nrf2 (#AF0639, Affinity, 1:1000), Keap-1 (#J1022, SANTA, 1:1000), HO-1 (#HA721854, HUABIO, 1:2000), P38 (#9212S, CST), Phospho-p38 (#Sc-166182, SANTA, 1:500), JNK (#9252S, CST, 1:1000), Phospho-JNK (#9255S, CST), Erk1/2 (#AF6240, Affinity, 1:1000), Phospho-Erk1/2 (#4370S, CST, 1:2000), Erk1(#F1152, Selleck, 1:1000), HRP secondary antibody (#GB23301, Servicebio), fluorescent secondary antibody 488 (#S0017, Affinity, 1:300). Ki67 (#A200018, ABclonal Company, 1:500), Monolith (Nano Temper, Germany), PowerPac HC protein electrophoresis apparatus, transfer membrane apparatus, chemiluminescence gel imaging system (Bio-rad, USA).

### 2.3 Western blot

A549 cells and H1975 cells were seeded in 6-well plates at a density of 2×10^5^ cells per well. Then, 1% culture medium containing TGF-β1 was added separately with or without Abrine (10-40 μM) (14) or Sch772984 (20 nM) (15) for 48 hours. 30 μg of protein were separated on an SDS-polyacrylamide gel and electrophoresed at 110 V for 1 h. The gel was transferred to a PVDF membrane at 100V for 2 hours and then blocked in 5% skimmed milk for 2 hours. Subsequently, the primary antibody was incubated for 12–18 hours, the secondary antibody was incubated for 2 hours, and finally, the chemiluminescence detection kit was prepared to uniformly cover the band with the imaging solution (A solution: B solution=1:1). Imaging was performed using a Bio−Rad imaging system, and the protein band data were processed using Image Lab. If it was an animal tissue protein sample, the lung tissue was cut with scissors, and the tissue lysis buffer was added to the lung tissue at a ratio of 1:9 (10 mg tissue: 90 µL lysis buffer). The tissue lysate (RIPA strong: Cocktail: PMSF = 100:1:1) was ground in a tissue grinder, centrifuged to obtain the supernatant for protein quantification, and the subsequent operations remained unchanged.

For all figures containing quantified Western blot results, band intensities were analyzed by densitometry using Image Lab software; For phosphorylated proteins, the phosphorylation level was calculated as the ratio of the phosphorylated protein signal to that of its corresponding total protein, i.e., the phosphor/total protein ratio; For normalization of total protein expression, β-actin was used as the internal loading control. The relative expression level of each target protein was calculated by dividing the gray value of the target protein band by that of the β-actin band; All Western blot quantification was based on three independent experiments, and the quantified results are presented as relative expression levels.

### 2.4 Wound healing

A549 cells and H1975 cells were seeded in 6-well plates at a density of 2×10^5^ cells per well and incubated overnight. A 200μL pipette was used to scratch the cells, and then they were washed three times with PBS. Subsequently, in the 1% culture medium containing TGF-β1, the cells were treated with or without Abrine (10–40 μM) or Sch772984 (20 nM) for 48 hours. Wound healing was observed and photographed under a microscope. Cell migration rates were calculated by analyzing migration distances using Image J software. Cell migration distance: This was determined by comparing the scratch distance at the initial time (0 h) with the scratch distance at the subsequent observation time (48 h). Cell migration rate = (scratch distance at 0 h -scratch distance at 48 h)/scratch distance at 0 h ×100%, the experiment was repeated three times.

### 2.5 Transwell assay

A549 cells or H1975 cells were starved in serum-free medium for 1h. In the cell invasion test, Matrigel (1:8, diluted with 1% medium) was added to the chamber for 1 hour. In addition, cells were seeded at a density of 2.5×10^4^ cells/mL into the upper chamber (250μL 1% medium per well) and the lower chamber containing 500μL complete RPMI-1640 medium supplemented with 10% FBS, the experiment was repeated three times. After co-incubation of Abrine with A549 cells or H1975 cells for 48 hours, the A549 or H1975 cells in the upper chamber were fixed with 4% paraformaldehyde (PFA), stained with crystal violet, and then the A549 or H1975 cells on the upper chamber side were wiped off with a cotton swab. Pictures were taken with an inverted microscope, and A549 cells or H1975 cells attached to the bottom of the upper chamber membrane were observed and counted. ImageJ was used to calculate the mobility, and the Image was imported into ImageJ to open. The image type was set as image-type-8bit, and the threshold was adjusted to ensure that each cell became black to avoid empty inside the cell. analyze-particles, size, select outlines, select display results, exclude on edges and summarize; A bar graph was drawn based on the count values calculated by summarize.

### 2.6 Immunofluorescence staining

A549 cells or H1975 cells (density: 2.5x10^4^ per well) were seeded in confocal culture dishes and incubated overnight. A549 cells were pretreated with Abrine (40 μM) for 4 hours and then co-treated with or without TGF-β1 for 48 hours. Next, the cells were fixed with 4% PFA for 15 minutes, permeabilized with 0.2% Triton X for 15 minutes, and blocked with 5% BSA for 30 minutes. Then, primary antibodies diluted in 5% BSA were incubated with the cells overnight, followed by staining with AF488 secondary antibodies for 2 hours and DAPI for 15 minutes. Fluorescence images were captured using a confocal laser scanning microscope.

### 2.7 Molecular docking

The three-dimensional spatial conformation data of Erk1 protein (protein number: P27361) was obtained from the Uniprot database. Using the PyMol software, water molecules and the original ligand were removed. Subsequently, with Erk1 proteins as receptors and Abrine molecule as the ligand, the active sites for molecular docking were determined based on the coordinates of the ligand in the target protein complex. Molecular docking was performed using AutoDock Vina, and relevant graphics were generated using PyMol.

### 2.8 Cell thermal migration assay

HEK293T cells were seeded in large dishes, with 2×10^6^ cells per well. After the cells grew fully, they were washed twice with PBS. Then, each well was lysed with 200 μL of lysis buffer. The lysates scraped off from each well were evenly divided into two portions and incubated with dimethyl sulfoxide (DMSO) or Abrine (40 μM) on ice for 1 hour. Subsequently, the samples were centrifuged at 15000 rpm for 25 minutes at 4 °C. The supernatants were evenly divided into 7 portions and heated at different temperatures (36, 40, 44, 48, 52, 56, 60 °C) for 3 minutes, then cooled at room temperature for 30 seconds, and subjected to protein immunoblotting experiments.

### 2.9 Drug affinity response target stability experiment

After HEK293T cells were inoculated into the petri dish, the supernatant was removed, washed with PBS, lysis buffer was added, and the mixture was incubated at 4 °C on ice for 15 minutes. The lysate was collected into a 1.5 mL EP tube, centrifuged at 15,000 rpm for 25 minutes at 4 °C, and the supernatant was collected. Protein quantification was performed using the BCA method. Protein samples and different concentrations of Abrine were incubated at 4 °C overnight, then Pronase E was added at a protein-protease ratio of 200:1 for digestion. Finally, the expression level of ERK protein was analyzed by Western blot to study the binding ability of Abrine to ERK protein and its effect on protein stability.

### 2.10 B16-F10 lung metastasis model mouse model

SPF-grade C57BL/6J male mice, 6–7 weeks old, weighing 18-22g, were provided by Guangdong Weitong Lihua Laboratory Animal Technology Co., Ltd. with the animal license number: SCXK (Yue) 2022-0063. Logarithmically growing and well-conditioned B16-F10 melanoma cells were digested with trypsin, the cell count was adjusted to adjust the cell concentration, and B16-F10 cells were inoculated at a ratio of 1×10^6^ cells per mouse into the tail vein of the mice, except for the blank control group. melanoma cells traveled through the bloodstream to the lungs and formed metastatic nodules (16). Randomly divided mice into the model group, Abrine (20 mg/kg) group, Abrine (40 mg/kg) group, Gefitinib (40 mg/kg) group (15), Sch772984 (12.5 mg/kg) group, and the Abrine (40 mg/kg) and Sch772984 combination group. There were 10 mice in each group. Administration started on the 4th day after modeling. Abrine was dissolved in normal saline and intraperitoneally injected once a day; Gefitinib was intragastrically administered once a day, and Sch772984 was intraperitoneally injected twice a day, with continuous administration for 17 days.

### 2.11 HE staining

The heart, liver, spleen, lung, kidney and brain tissues were fixed with 4% paraformaldehyde (PFA) for sectioning and staining with HE. Tissue slides were prepared to facilitate the observation of pathological changes of the tissues under a light microscope.

### 2.12 IHC

Paraformaldehyde-fixed, paraffin-embedded tissue samples were dewaxed with xylene and rehydrated using a graded ethanol series. For heat-induced epitope retrieval (HIER), sections were incubated in EDTA buffer (pH 8.0) and heated to near boiling in a microwave or pressure cooker for 10–20 min, then cooled naturally to room temperature. Endogenous peroxidase activity was blocked with 0.3% hydrogen peroxide for 15 min. After serum blocking, sections were incubated with the appropriate primary antibody at 4 °C overnight, followed by incubation with the corresponding secondary antibody. Color development was performed using AEC staining solution according to the manufacturer’s instructions. Stained sections were observed and photographed under a light microscope.

### 1.13 Comparison of lung wet weight and lung coefficient

After removing the lung tissue, quickly weigh the lung to obtain the wet weight of the lung; calculate the lung coefficient, where the lung coefficient = lung wet weight/body weight. By comparing the lung wet weight and lung coefficient, the degree of lung injury and the effect of drug treatment can be quantitatively analyzed.

### 2.14 MTT assay

A549 cells or H1975 cells in logarithmic growth phase were collected, and the cell density was adjusted to 0.5×10^4^ cells/well. Then, the cells were seeded into 96-well plates and continuously cultured for 18 hours to make the cells adhere to the wall. Different concentrations of TGF-β1 (0, 2.5, 5, 10mg/kg) or Abrine (0, 10, 20, 40μM) were added into the corresponding Wells, and the cells were cultured for 48 hours. MTT solution at a concentration of 5 mg/mL was diluted 10-fold with 1% fetal bovine serum. The cell culture medium containing the drug in the 96-well plate was discarded, and MTT diluent was added. The supernatant was discarded, and 100 μL dimethyl sulfoxide (DMSO) was added to each well, and the mixture was evenly mixed by shaking at low speed for 10 min-15 min. The microplate reader was set at 490 nm to detect the absorbance (OD value), and the effect of cell survival rate was calculated. MTT assay statistical methods: The DMSO group was used as the standard group, the average value of the DMSO group was taken, and the quantitative results were obtained for each measured well/DMSO group. Experiments were repeated 3 times.

### 2.15 Statistical analysis

Statistical analysis was performed using GraphPad Prism 10.1.2 software. All measurements were repeated at least three times. Statistical significance was determined using one-way ANOVA and Dunnett’s multiple comparison for inter-group differences. No significant differences were *P*>0.05; ^*^P < 0.05; ^**^P < 0.01; ^***^P < 0.001; ^#^*P* < 0.05; ^##^*P* < 0.01; ^###^*P* < 0.001.

## 3 Results

### 3.1 Abrine suppresses TGF-β1-induced EMT, migration, and invasion in NSCLC cells

**Figure 2:**
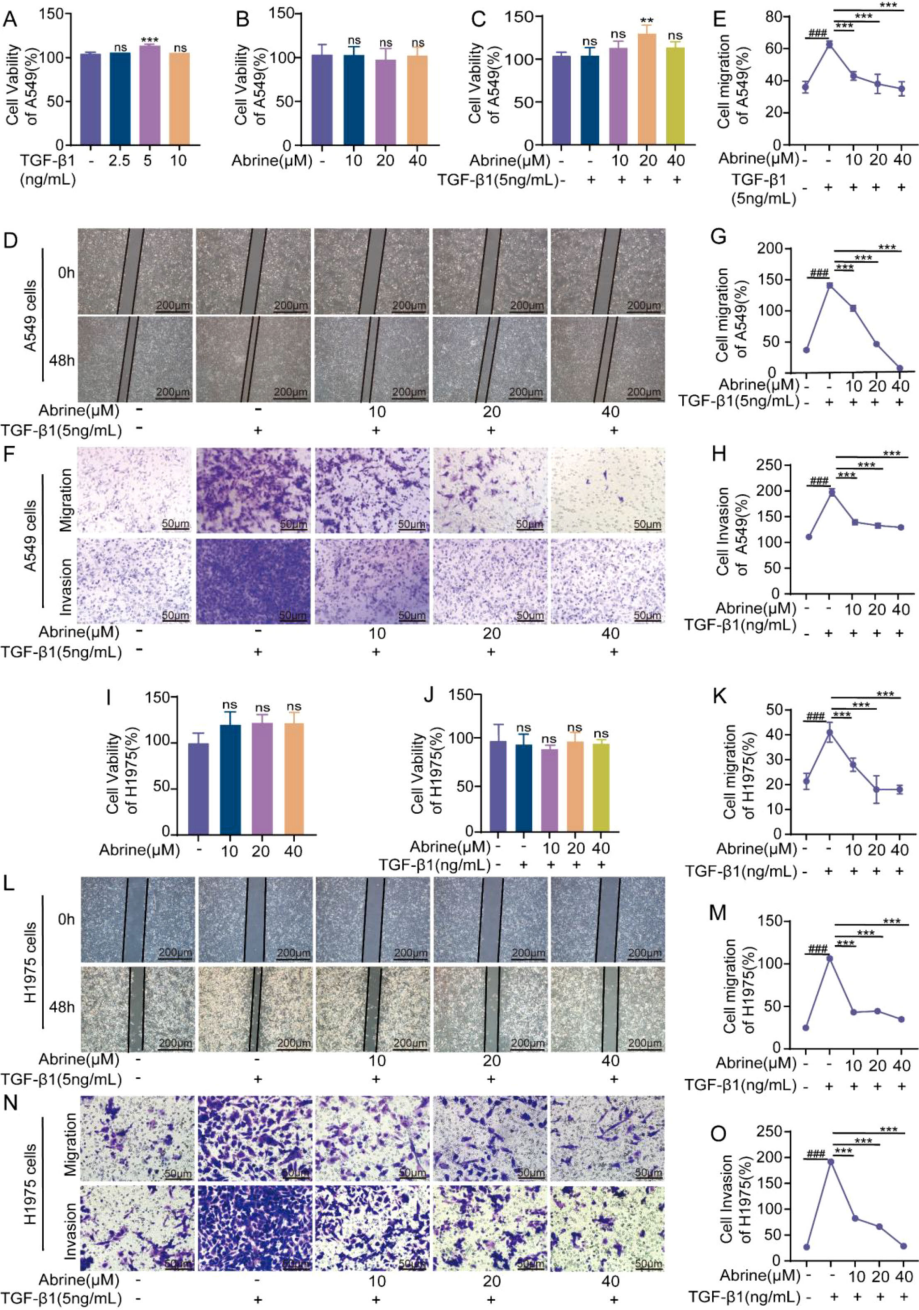
Abrine suppresses TGF-β1-induced EMT, migration, and invasion in NSCLC cells. **(A)** TGF-β1 (2.5–10 ng/mL) on the cytotoxicity of A549 cells detected by MTT method, n=3; **(B, I)** The cytotoxicity of A549 and H1975 cells after treatment with Abrine (10-40 μM) for 48 hours measured by MTT method, n=3; **(C, J)** Pre-treatment of A549 and H1975 cells with Abrine for 4 hours, followed by culturing with TGF-β1 (5 ng/mL) for 48 hours, n=3; **(D-E, K-L)** Cell scratch assay to detect the effect of Abrine on the migration ability of TGF-β1induced A549 and H1975 cells, scale: 100 μm, n=3; **(F-H, M-O)** Transwell assay to detect the effect of Abrine on the migration and invasion abilities of TGF-β1-induced A549 and H1975 cells, scale: 50 μm, n=3. ^###^*P* < 0.001 vs blank control group, ^***^P < 0.001 vs TGF-β1 model group, ^**^P < 0.01 vs TGF-β1 model group, ns no statistical difference.

TGF-β1 plays a crucial role in cancer progression and is a classic EMT inducer. It mainly promotes tumor progression by enhancing EMT and creating a favorable tumor microenvironment, rather than directly promoting cell proliferation (17, 18). Co-incubation of A549 and H1975 cells with abrine (10, 20, 40 μM) for 48 hours produced no cytotoxic effects, as confirmed by MTT assays (**Figures 2B, I**). Treatment of A549 cells with TGF-β1 (2.5, 5, 10 ng/mL) for 48 hours revealed that 5 ng/mL achieved optimal EMT induction (**Figure 2A**). Subsequent co-treatment with abrine (10-40 μM) for 48 hours did not affect cell viability in either cell line (**Figures 2C, J**). Wound-healing assays demonstrated that TGF-β1 markedly enhanced the migratory capacity of both A549 and H1975 cells after 48 hours, whereas abrine significantly suppressed TGF-β1-induced migration (**Figures 2D, E, L, K**). Similarly, Transwell invasion assays showed that TGF-β1 greatly increased cell invasiveness, which was effectively inhibited by abrine (**Figures 2F-H, M-O**). Collectively, these findings indicate that abrine robustly inhibits TGF-β1-induced migration and invasion in both A549 and H1975 cells, highlighting its potential as an EMT-targeting agent in lung cancer therapy.

### 3.2 Abrine reverses TGF-β1-induced EMT-related markers in NSCLC cells

**Figure 3:**
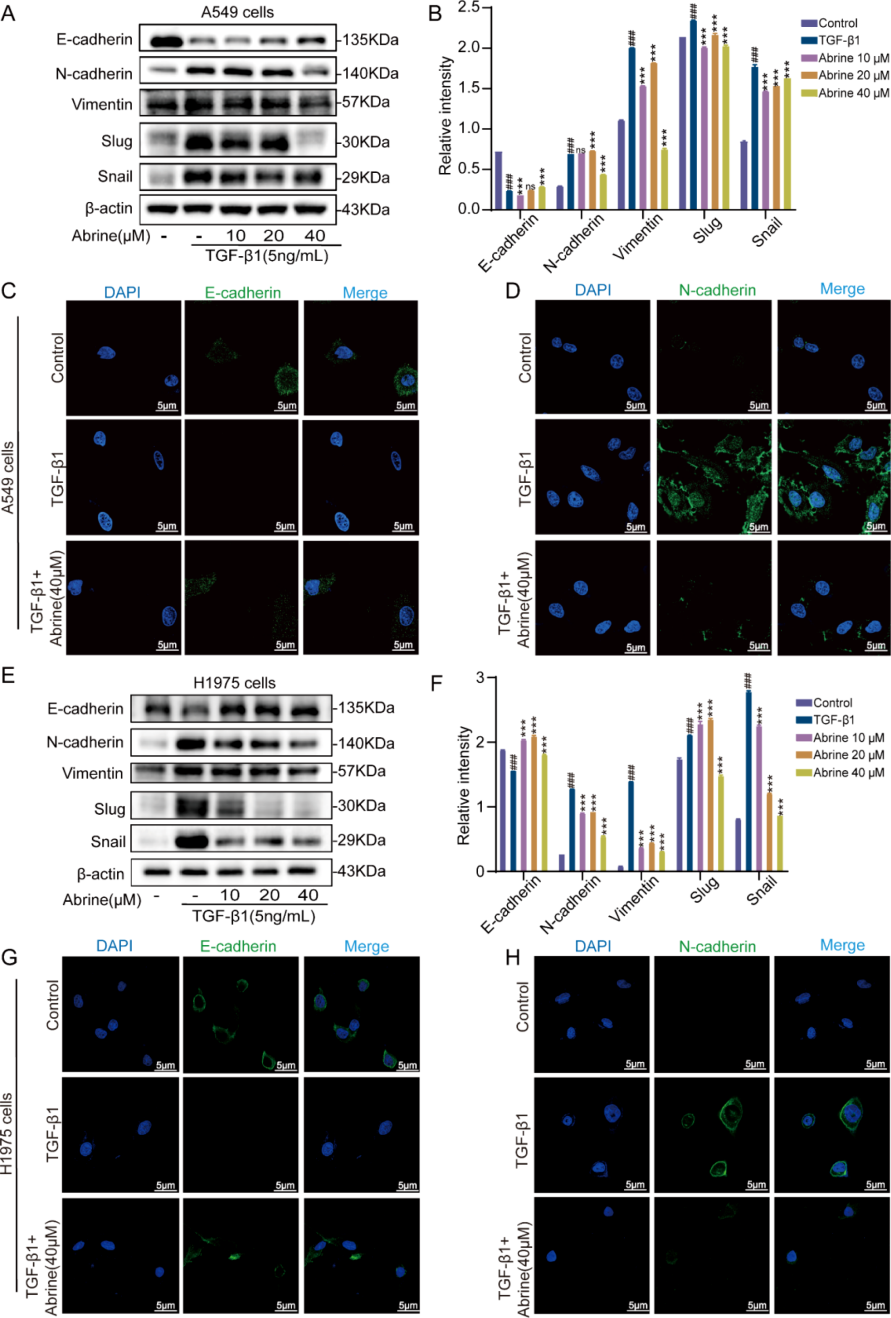
Abrine reverses TGF-β1-induced EMT-related markers in NSCLC cells. **(A, B, E, F)** Western blot analysis of Abrine’s effect on protein expression of EMT markers in A549 and H1975 cells induced by TGF-β1, n=3; **(C, D, G, H)** Immunofluorescence method was used to detect the protein expressions of E-cadherin and N-cadherin in A549 and H1975 cells induced by TGF-β1, scale: 5μm, n=3; ^###^*P* < 0.001 vs blank control group, ^***^P < 0.001 vs TGF-β1 model group, ^**^P < 0.01 vs TGF-β1 model group, ns no statistical difference.

After 48 hours TGF-β1 induction, western blot analysis of EMT-related proteins revealed that TGF-β1 markedly reduced the expression of the epithelial marker E-cadherin while elevating the mesenchymal markers N-cadherin and Vimentin, as well as the transcription factors Slug and Snail. Treatment with Abrine significantly restored E-cadherin expression and downregulated N-cadherin, Vimentin, Slug, and Snail levels (**Figures 3A, B, E, F**). Immunofluorescence staining corroborated these results, after TGF-β1 treatment, green fluorescence of E-cadherin markedly decreased, while N-cadherin fluorescence intensified. Co-treatment with abrine effectively reversed these fluorescence patterns (**Figures 3C, D, G, H**). Together, these findings indicate that Abrine effectively enhances epithelial marker expression while suppressing mesenchymal markers and EMT-associated transcription factors, thereby reversing TGF-β1-induced EMT in A549 and H1975 cells.

### 3.3 Abrine modulates MAPK and Nrf2 signaling pathways to inhibit TGF-β1-induced EMT in NSCLC cells

**Figure 4:**
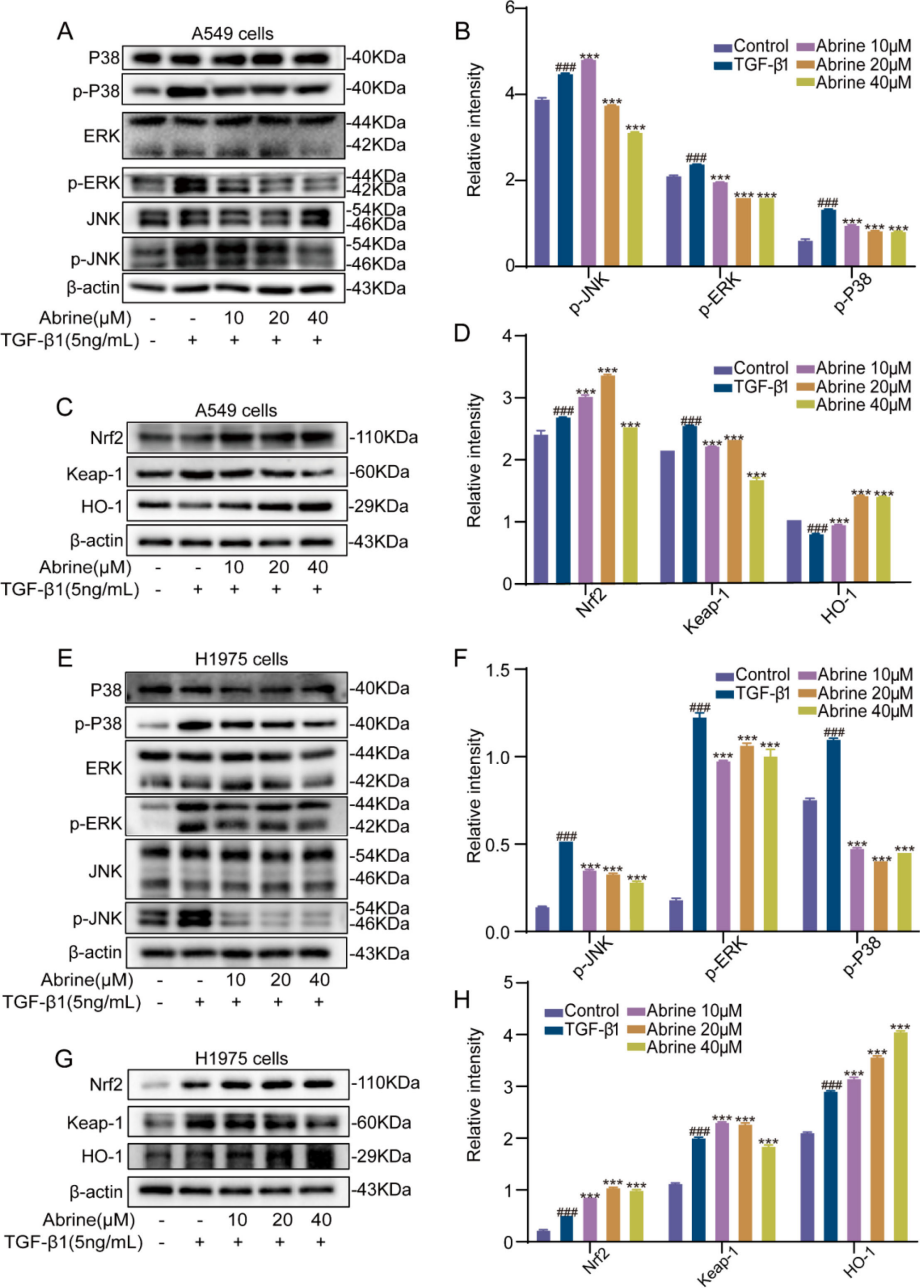
Abrine modulates MAPK and Nrf2 signaling pathways to inhibit TGF-β1-induced EMT in NSCLC cells. **(A, B, E, F)** Western blot was used to detect the changes in P38, p-P38, JNK, p-JNK, ERK, and p-ERK proteins in A549 and H1975 cells induced by TGF-β1 in the presence of Abrine, n=3; **(C, D, G, H)** Western blot was used to detect the expression of Nrf2, Keap-1, and HO-1 proteins in A549 and H1975 cells induced by TGF-β1 in the presence of Abrine, n=3; ^###^*P* < 0.001 vs blank control group, ^***^P < 0.001 vs TGF-β1 model group, ^**^P < 0.01 vs TGF-β1 model group.

MAPKs play a crucial regulatory role in the EMT process through phosphorylation and activation of specific transcription factors (19). The MAPKs/ERK pathway is a key regulatory factor for EMT (20, 21). Activated ERK moves to the nucleus and regulates the activities of various transcription factors such as Snail and Slug, which in turn regulate genes related to EMT (22, 23). As shown in **Figures 4A and E**, treatment with TGF-β1 (5 ng/mL) markedly increased the phosphorylation levels of MAPK family proteins-Erk1/2, p38, and JNK-in A549 and H1975 cells, indicating pathway activation. Co-treatment with Abrine (10, 20, 40 μM) significantly attenuated TGF-β1-induced phosphorylation of these proteins, with statistically significant reductions (**Figures 4B, F**). Furthermore, after 48 hours co-treatment with TGF-β1 (5 ng/mL) and Abrine (10, 20, 40 μM), western blot analysis revealed that abrine decreased Keap-1 protein expression while upregulating Nrf2 and HO-1 levels (**Figures 4C, D, G, H**). Since Keap-1 regulates Nrf2 protein upregulation through ubiquitin-mediated degradation, and HO-1 is a downstream effector of Nrf2, these findings suggest that Abrine may regulate the Nrf2/HO-1 antioxidant pathway by inhibiting Keap-1. Together, these results indicate that Abrine suppresses EMT by inhibiting MAPK pathway activation and regulating the Nrf2/Keap-1/HO-1 signaling axis, thereby exerting dual regulation on oxidative stress and metastatic signaling in NSCLC cells.

### 3.4 Abrine directly binds to ERK

**Figure 5:**
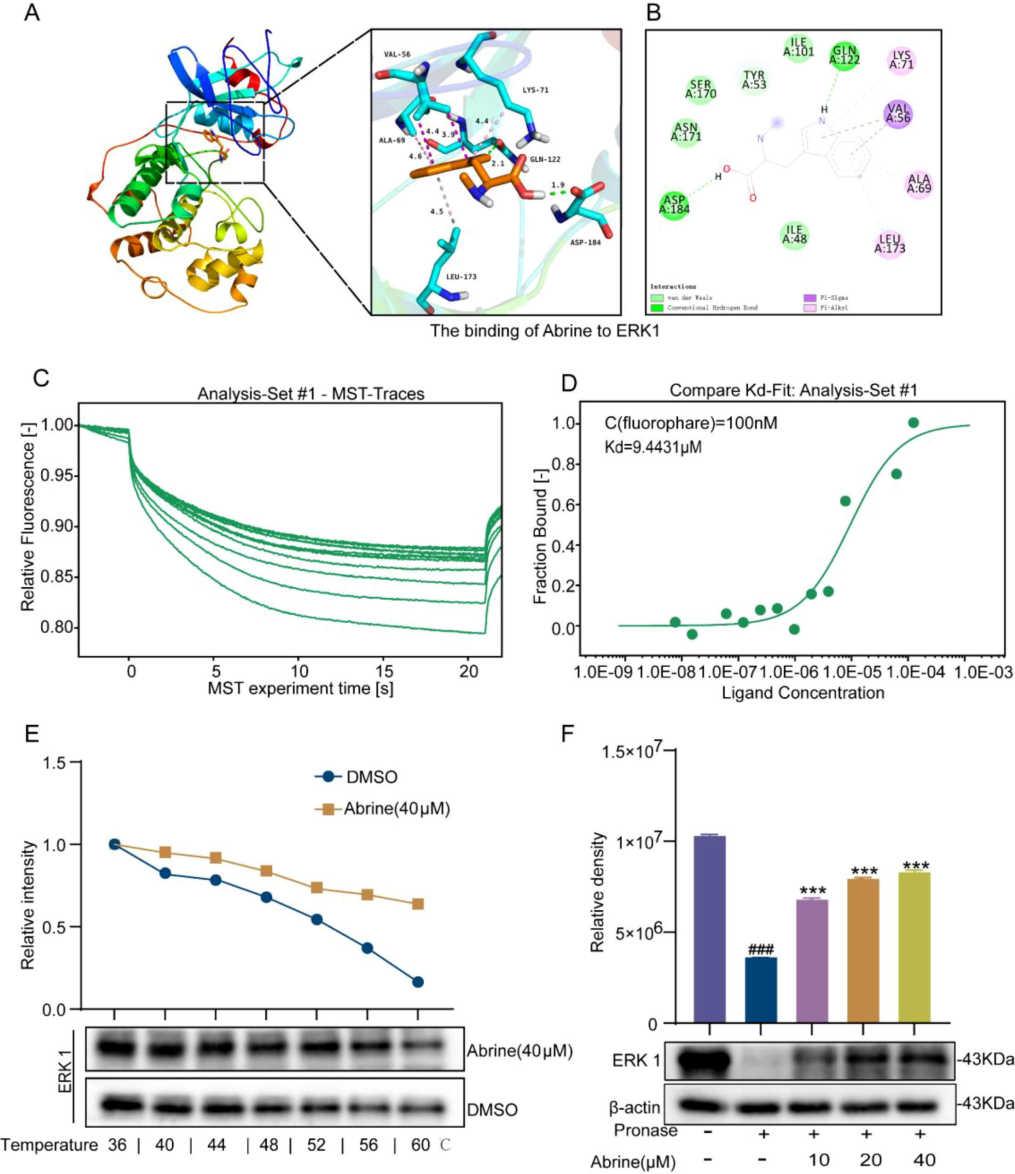
Abrine directly binds to ERK. **(A, B)** The results of the molecular docking of Abrine with ERK1; **(C, D)** The fitting curve of the dissociation constant for the Abrine-ERK protein interaction, n=3, Dissociation constant (Kd): 9.4431 µM (with a confidence interval of 4.31–20.7 µM), R²=0.98, Signal-to-Noise=11.5; **(E)** The cell thermal migration experiment verified the binding characteristics of Abrine with ERK, n=3; **(F)** The target stability experiment of the drug affinity reaction detected the stability of the binding of Abrine with ERK, n=3. ^###^*P* < 0.001 vs the blank control group, ^***^P < 0.001 vs the TGF-β1 model.

It has been proven through research that the improper activation of the ERK signaling pathway is associated with lung cancer, and this pathway is crucial for inducing the progression of EMT (24). Molecular docking showed that Abrine could bind to ERK1 with a binding energy of −7.0 kcal/mol, with predicted interactions involving residues ASP184, GLN122, LYS71, VAL56, ALA69, and LEU173 (**Figures 5A, B**). We quantitatively analyzed the binding affinity between Abrine and ERK using microscale thermophoresis (MST). At 25°C, the dissociation constant (Kd) was 9.4431 μM, indicating a moderate binding affinity between Abrine and ERK (**Figures 5C, D**). MST assay result supported that ERK was identified as a key potential target of Abrine. To experimentally validate this interaction, a cellular thermal shift assay (CETSA) was conducted. The results demonstrated that under heat stress conditions, Abrine markedly increased the thermal stability of ERK1, indicating direct compound-protein engagement (**Figure 5E**). Consistently, the drug affinity responsive target stability (DARTS) assay showed that Abrine reduced the susceptibility of ERK1 to protease digestion, further supporting target protection upon binding (**Figure 5F**). Taken together, these orthogonal assays provide convergent evidence that Abrine directly binds to ERK and engages this protein at the molecular level. Based on these results, we consider ERK to be a likely direct target of Abrine.

### 3.5 Abrine targets on ERK to reverse TGF-β1-induced EMT in NSCLC cells

**Figure 6:**
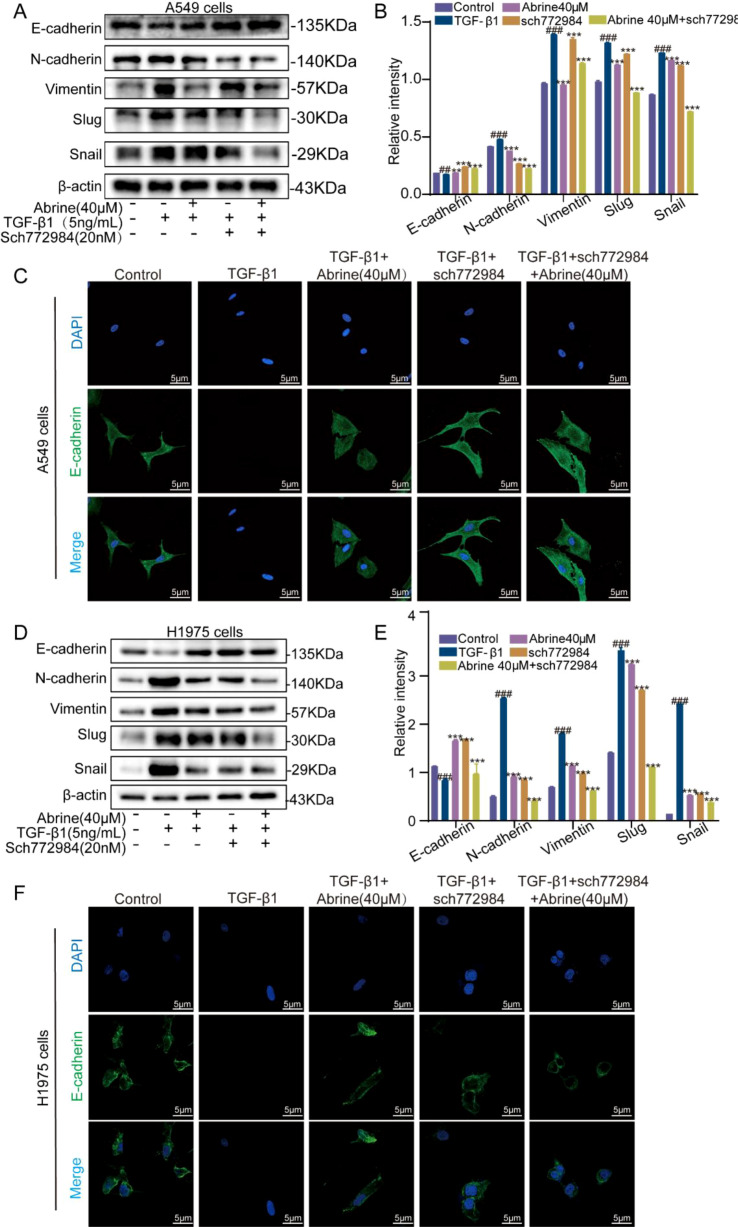
Abrine targets on ERK to reverse TGF-β1-induced EMT in NSCLC cells. **(A, B, D, E)** Western blot was used to detect the effects of Abrine and Sch772984 on the EMT-related markers in A549 and H1975 cells induced by TGF-β1, n=3; **(C, F)** Immunofluorescence was used to detect the effects of Abrine and Sch772984 on the expression of E-cadherin protein in A549 and H1975 cells, scale: 5μm, n=3. ^###^*P* < 0.001 vs blank control group, ^***^P < 0.001 vs TGF-β1 model group, ^**^P < 0.01 vs TGF-β1 model group, ns no statistical difference.

To further validate the role of ERK in TGF-β1-induced EMT, the specific ERK1/2 inhibitor SCH772984 was applied to A549 and H1975 cells. As shown in **Figures 6A, B** and 6D-E, TGF-β1 treatment markedly downregulated E-cadherin and upregulated N-cadherin, Vimentin, Slug, and Snail, confirming EMT activation. Treatment with Abrine (40 μM) or SCH772984 (20 nM) alone effectively reversed these changes, restoring epithelial characteristics and suppressing mesenchymal marker expression. Notably, combined treatment with Abrine and SCH772984 produced a more pronounced reversal effect than either agent alone. Consistent with these results, immunofluorescence analysis revealed that TGF-β1 markedly reduced E-cadherin-associated green fluorescence, while administration of Abrine, SCH772984, or their combination significantly restored fluorescence intensity (**Figures 6C, F**). Together, these findings demonstrate that Abrine exerts its EMT-inhibitory effects at least partly through ERK signaling modulation, and co-inhibition of ERK amplifies its suppressive impact on TGF-β1-induced EMT in NSCLC cells.

### 3.6 Abrine targets ERK to reverse TGF-β1-induced EMT in NSCLC cells via MAPK and Nrf2 signaling pathways

**Figure 7:**
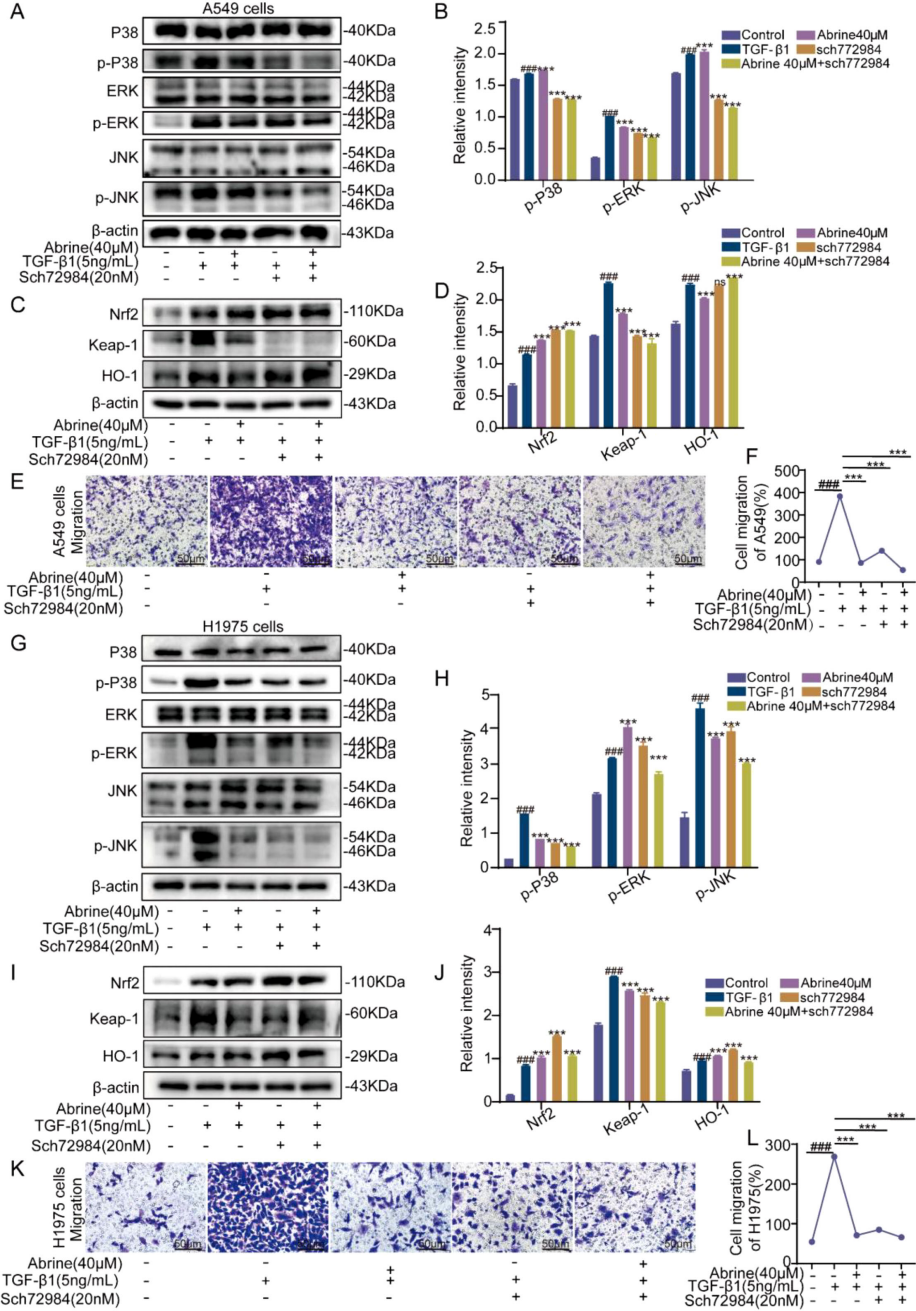
Abrine targets ERK to reverse TGF-β1-induced EMT in NSCLC cells via MAPK and Nrf2 signaling pathways. **(A, B, G, H)** Western blot was used to detect the effects of Abrine and Sch772984 on the key proteins of the MAPKs signaling pathway in A549 and H1975 cells induced by TGF-β1, n=3; **(C, D, I, J)** Western blot was used to detect the effects of Abrine and Sch772984 on the Nrf2/Keap-1/HO-1 signaling pathway in A549 and H1975 cells induced by TGF-β1, n=3; **(E, F, K, L)** Transwell assay was used to detect the effects of Abrine and Sch772984 on the migration ability of A549 and H1975 cells induced by TGF-β1, scale: 50 μm, n=3. ^###^*P* < 0.001 vs blank control group, ^***^P < 0.001 vs TGF-β1 model group, ^**^P < 0.01 vs TGF-β1 model group, ns no statistical difference.

Compared with the blank control group, TGF-β1 induction (5 ng/mL, 48 hours) markedly elevated the phosphorylation levels of p38, ERK, and JNK in both A549 and H1975 cells. Treatment with Abrine (40 μM) or SCH772984 (20 nM) effectively reduced the phosphorylation of these MAPK family proteins (**Figures 7A, B, G, H**), indicating that Abrine may suppress EMT by modulating the TGF-β1-mediated MAPK signaling cascade. Western blot analysis further showed that both Abrine and SCH772984 treatment affected the Nrf2/Keap-1/HO-1 signaling pathway, as shown by increased Nrf2 and HO-1 expression and decreased Keap-1 expression (**Figures 7C, D, I, J**), suggesting a possible modulation of antioxidant defense mechanisms. Moreover, transwell migration assays demonstrated that Abrine, SCH772984, and their combination all significantly inhibited TGF-β1-induced cell migration in both A549 and H1975 cells after 48 hours (**Figures 7E, F, K, L**). Collectively, these findings indicate that ERK plays a pivotal regulatory role in Abrine’s reversal of TGF-β1-induced EMT, acting through dual modulation of the MAPK and Nrf2/Keap-1/HO-1 signaling pathways to inhibit cell migration and mesenchymal transformation.

### 3.7 Abrine combined with ERK inhibition suppresses lung metastasis in B16-F10 mice model

**Figure 8:**
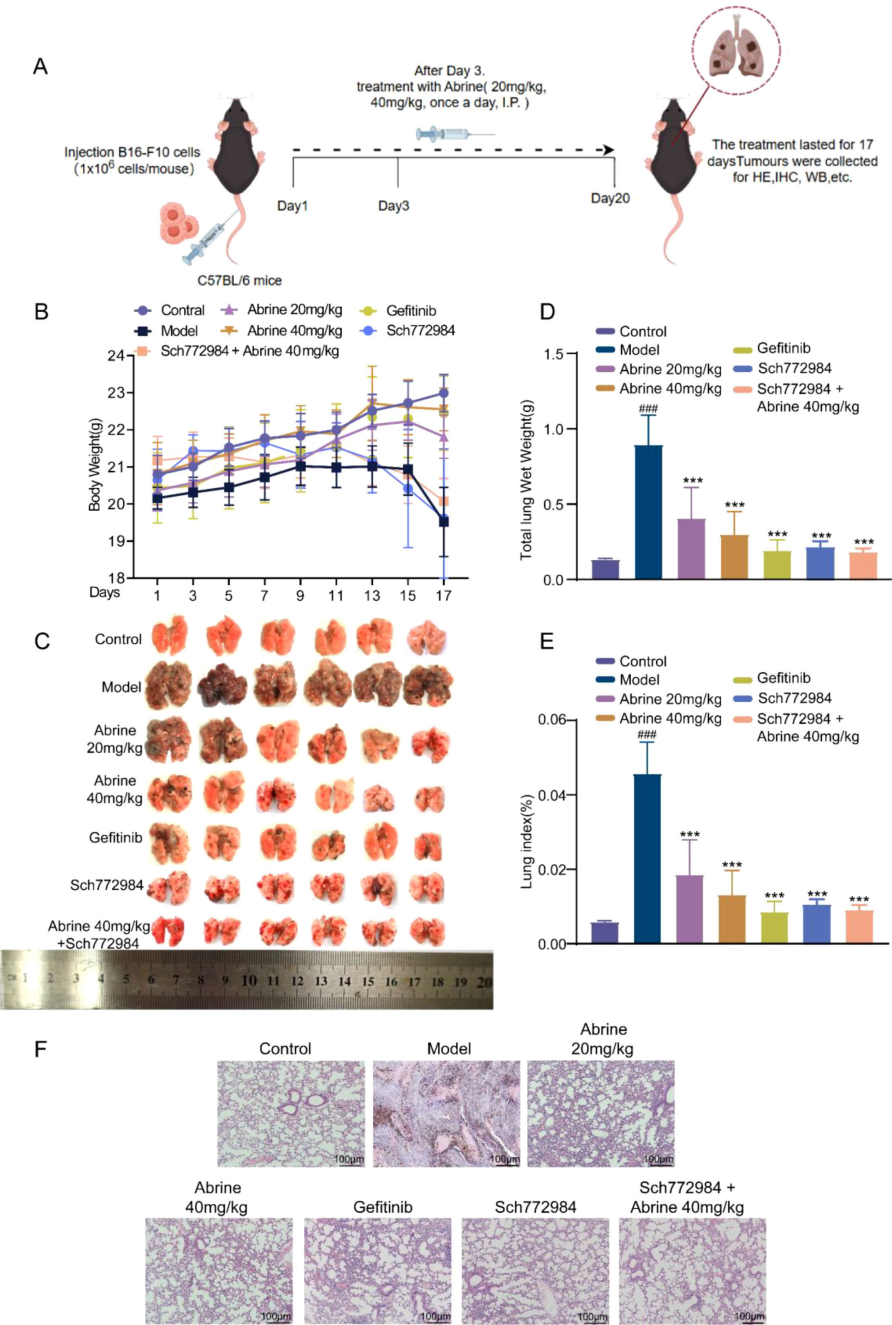
Abrine combined with ERK inhibition suppresses lung metastasis in B16-F10 mice model. **(A)** Model establishment and administration method for B16-F10 lung metastasis in mice; **(B)** The effect of Abrine on the body weight of B16-F10 lung metastasis mice, n=6 per group; **(C)** Observation of the effect of Abrine on lung metastasis in B16-F10 lung metastasis mice, n=6 per group; **(D)** The effect of Abrine on the total lung wet weight of B16-F10 lung metastasis mice, n=6 per group; **(E)** The effect of Abrine on the lung index of B16-F10 lung metastasis mice, Lung index = Lung weight/Body weight × 100%. **(F)** The effect of Abrine on the lung tissue of B16-F10 lung metastasis model mice (scale=100μm). ^###^*P* < 0.001 vs blank control group, ^***^P < 0.001 vs TGF-β1 model group, n=6 per group.

As shown in **Figure 8A**, the experimental schedule involved tail-vein injection of B16-F10 melanoma cells followed by daily intraperitoneal administration of Abrine, daily oral dosing of Gefitinib (positive control), and twice-daily intraperitoneal injection of the ERK1/2 inhibitor SCH772984 for a total of 17 days. Compared with the control group, mice in the model group exhibited significant body weight loss. In contrast, treatment with Abrine (at various doses), Gefitinib, SCH772984, or their combination notably improved body weight (**Figure 8B**). Pathological evaluation revealed severe melanoma lung metastasis in model mice, characterized by lung enlargement, increased metastatic nodules, elevated wet lung weight, and lung coefficient (**Figures 8C–E**). These lesions were markedly alleviated by all treatment regimens. Both Abrine and SCH772984 alone significantly reduced the number and size of metastatic nodules and decreased lung wet weight and coefficient, while the combined treatment achieved the most pronounced therapeutic effect. Further histological examination of lung tissues revealed extensive tumor metastasis, alveolar destruction, and severe pathological damage in the model group. In contrast, after 17 days of continuous administration, the Abrine, Gefitinib, SCH772984, and Abrine + SCH772984 treatment groups exhibited markedly improved lung architecture, with reduced tumor infiltration and attenuated tissue injury (**Figure 8F**). These results confirm that Abrine effectively suppresses pulmonary metastasis while exhibiting a favorable safety profile, and that co-administration with ERK inhibition enhances its therapeutic efficacy without inducing organ toxicity.

### 3.8 Abrine and ERK inhibition attenuate inflammation and tumor proliferation in B16-F10 mice model

**Figure 9:**
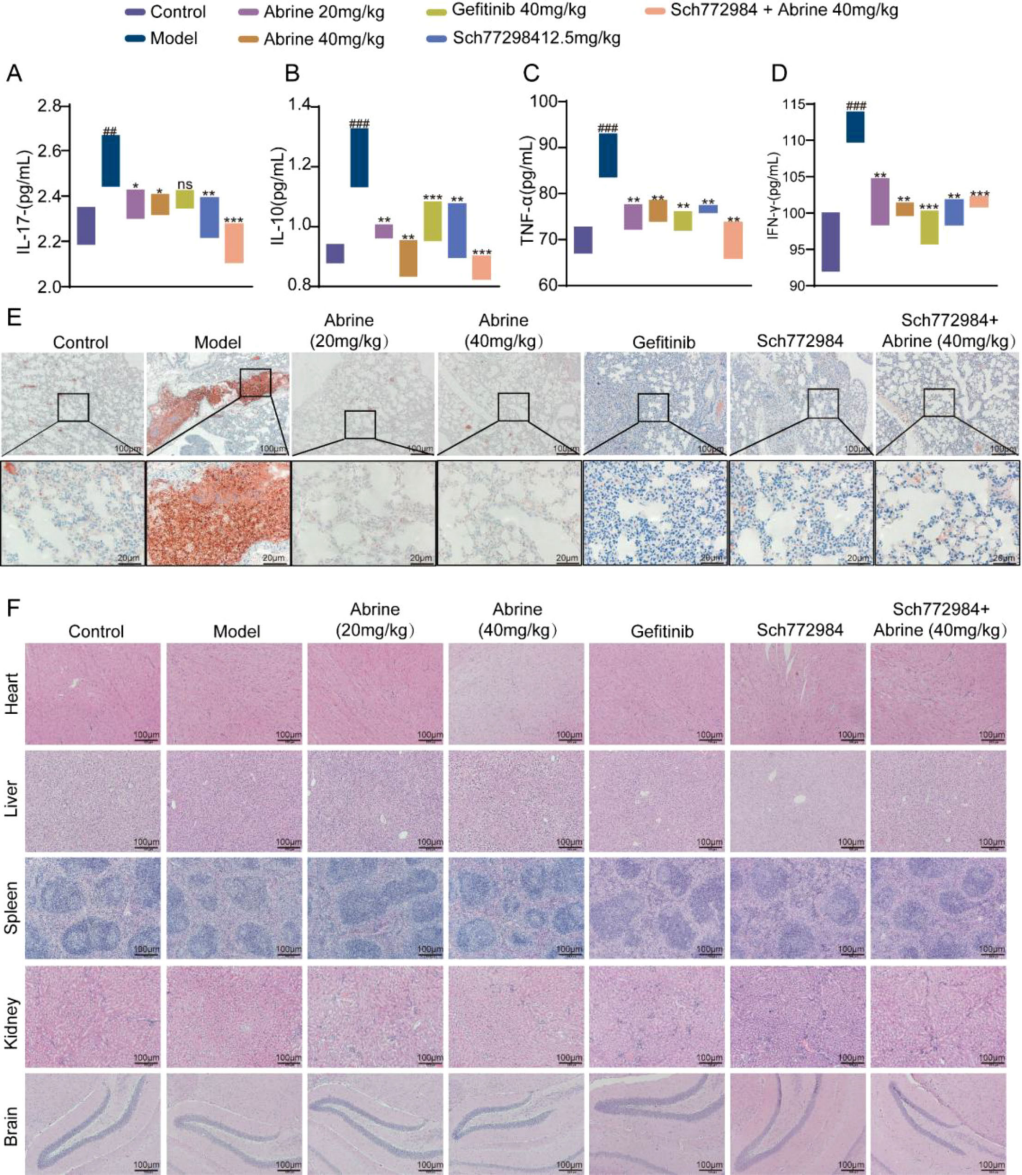
Abrine and ERK inhibition attenuate inflammation and tumor proliferation in B16-F10 mice model. **(A-D)** ELISA experiments were used to detect the levels of IL-17, IL-10, TNF-α and IFN-γ in the serum of B16-F10 lung metastasis model mice, n=6 per group. **(E)** IHC was used to detect the expression of Ki67 in the lung tissue of B16-F10 lung metastasis model mice, original scale: 100μm, magnified scale: 20μm. **(F)** HE staining was used to detect the effects of Abrine on the heart, liver, spleen, kidney and brain tissues of B16-F10 lung metastasis model mice; scale:100μm. ^###^*P* < 0.001 vs blank control group, ^***^P < 0.001 vs TGF-β1 model group, ^**^P < 0.01 vs TGF-β1 model group, ns no statistical difference.

As shown in **Figures 9A–D**, ELISA analysis of serum from B16-F10 lung metastasis mice revealed significantly elevated levels of IL-17, IL-10, TNF-α, and IFN-γ in the model group compared with controls, indicating strong inflammatory activation and immune dysregulation. Treatment with Abrine, Gefitinib, or Abrine combined with SCH772984 markedly reduced these cytokine levels, suggesting attenuation of systemic inflammation. Furthermore, immunohistochemical (IHC) staining for the proliferation marker Ki67 revealed markedly higher expression in metastatic lung tissues of model mice, whereas treatment with Abrine, Gefitinib, SCH772984, or their combination significantly suppressed Ki67 expression (**Figure 9E**). HE staining was performed on the heart, liver, spleen, kidney, and brain tissues of treated mice. No significant pathological alterations were observed compared with the control group, indicating no apparent systemic toxicity from any treatment ((**Figure 9F**). Collectively, these results demonstrate that Abrine alleviates inflammatory responses and inhibits tumor proliferation in lung metastasis, and that ERK pathway inhibition further enhances these therapeutic effects.

### 3.9 Abrine and ERK inhibition suppresses EMT to inhibit lung metastasis in B16-F10 mice model via MAPKs and Nrf2 pathways

**Figure 10:**
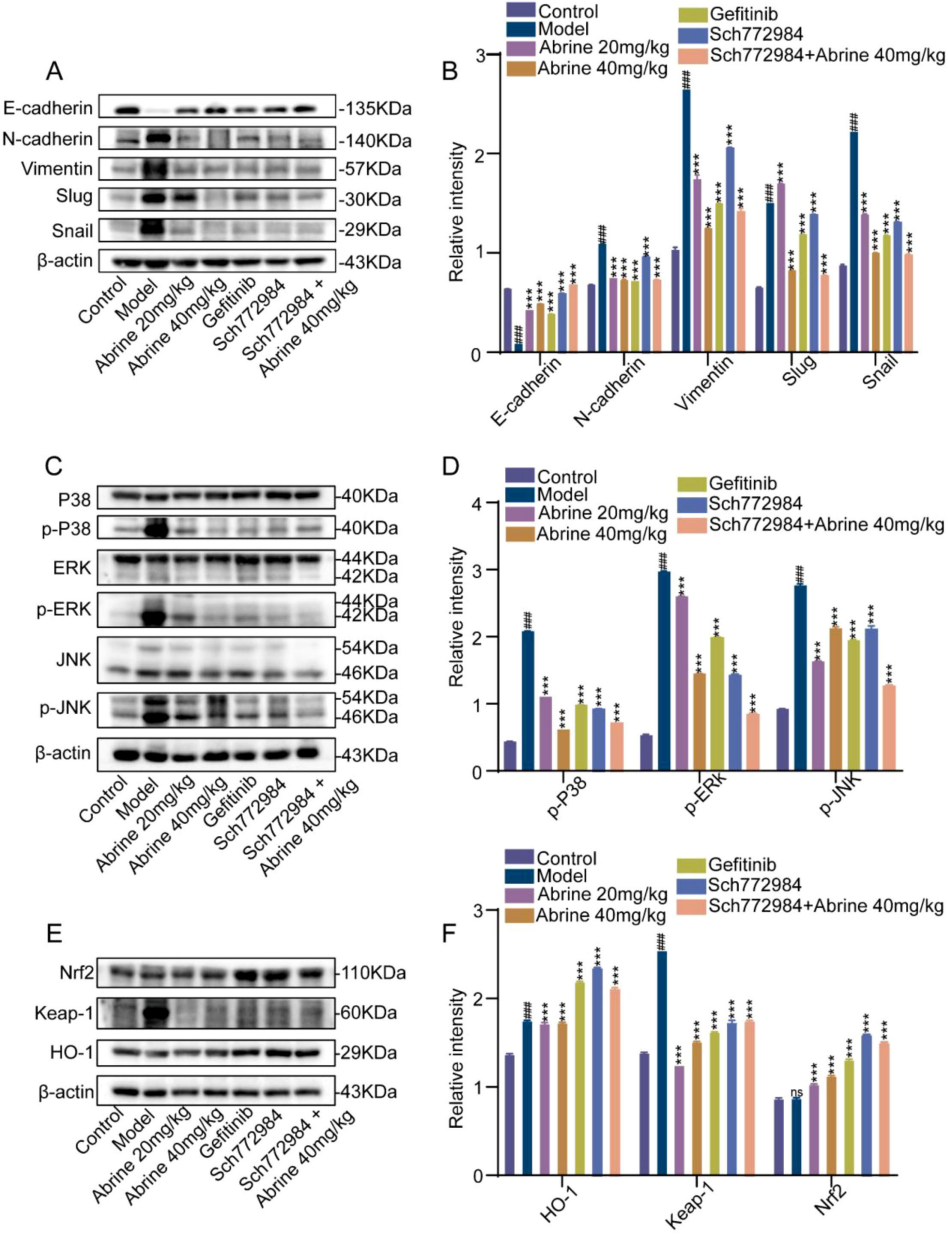
Abrine and ERK inhibition suppresses EMT to inhibit lung metastasis in B16-F10 mice model via MAPKs and Nrf2 pathways. **(A, B)** Western blot detected the effects of Abrine and Abrine combined with Sch772984 on EMT-related marker proteins in the lung tissues of B16-F10 lung metastasis mice, n=3; **(C, D)** Western blot detected the effects of Abrine and Abrine combined with Sch772984 on key proteins of the MAPKs signaling pathway in the lung tissues of B16-F10 lung metastasis mice, n=3; **(E)** Western blot detected the effects of Abrine and Abrine combined with Sch772984 on key proteins of the Nrf2/Keap-1/HO-1 signaling pathway in the lung tissues of B16-F10 lung metastasis mice, n=3; **(F)** is the statistical result of Figure E. *^###^P* < 0.001 vs blank control group, ^***^P < 0.001 vs TGF-β1 model group, ^**^P < 0.01 vs TGF-β1 model group, ns no statistical difference.

As shown in **Figures 10A, B**, compared with the blank control group, the model group exhibited a marked decrease in E-cadherin expression and significant increases in N-cadherin, Vimentin, Snail, and Slug levels, indicating activation of EMT. Treatment with Abrine, Gefitinib, SCH772984, or their combination effectively reversed these changes upregulating E-cadherin while downregulating N-cadherin, Vimentin, Snail, and Slug suggesting suppression of EMT. In **Figures 10C, D**, TGF-β1 stimulation led to elevated phosphorylation of p38, ERK1/2, and JNK in the model group, while treatment with Abrine, Gefitinib, or SCH772984 significantly reduced these phosphorylation levels, indicating inhibition of MAPK pathway activation. Furthermore, Western blot analysis of Nrf2/Keap-1/HO-1 signaling showed that Abrine, Gefitinib, SCH772984, and their combination suppressed Keap-1 dissociation from the Keap-1-Nrf2 complex, thereby increasing Nrf2 protein levels and enhancing expression of its downstream target HO-1 (**Figures 10E, F**). Collectively, these findings suggest that Abrine, alone or in combination with ERK inhibition, suppresses lung metastasis model by downregulating EMT-associated markers, inhibiting MAPK phosphorylation, and regulated the Nrf2/Keap-1/HO-1 antioxidant pathway.

## 4 Discussion

The core objective of this study is to elucidate how Abrine inhibits the epithelial-mesenchymal transition (EMT) and metastatic properties in non-small cell lung cancer (NSCLC) cells through the ERK/MAPK-related signaling pathways. Thus, NSCLC cell lines offer the most suitable and clinically relevant cellular system for mechanistic validation. Additionally, we used transforming growth factor-β1 (TGF-β1) to induce EMT and quantified the EMT markers and cellular functional behaviors through wound healing experiments and Transwell assays to detect the migration and invasion abilities of the cells (25–28). A549 and H1975 are mature non-small cell lung cancer (NSCLC) models related to EMT (29–31), and using two independent non-small cell lung cancer cell lines helps enhance the universality of our conclusions and makes them not limited by a single genetic background. Particularly, the H1975 cell line exhibits obvious EMT and mixed characteristics (32), which is of great value for evaluating EMT reversal and pathway targeting effects. Therefore, the selection of A549 and H1975 cell lines is to study the phenotypes (migration or invasion) related to metastasis and epithelial-mesenchymal transition (EMT) in non-small cell lung cancer (NSCLC), rather than simulating organ-specific lung colonization.

Furthermore, to evaluate lung colonization and metastatic burden at the organ level, we conducted an *in vivo* lung metastasis load experiment using the tail vein injection model of B16-F10. This model can generate quantifiable lung metastatic nodules (33). Thus, our experimental design distinguishes between two related but different issues. The *in vitro* experiments aim to regulate EMT and migration/invasion in non-small cell lung cancer cells (A549/H1975) by Abrine, clarifying its mechanism of action. The *in vivo* experiments utilize Abrine to reduce the lung metastasis load/colonization in the established lung nodule model (B16-F10) to prove its anti-metastasis efficacy at the organ level.

During the EMT process, the expression of epithelial marker E-cadherin decreases, while the expressions of mesenchymal markers N-cadherin, vimentin, and transcription factors Snail and Slug increase. This change directly promotes the transformation of tumor cells from the epithelial phenotype to the mesenchymal phenotype, enhancing their metastatic ability (34, 35). TGF-β1 is an important factor driving EMT (36). In this study, TGF-β1 was used to induce the occurrence of EMT, significantly enhancing the migration and invasion abilities of A549 and H1975 cells. After the intervention of Abrine, the results of cell scratch assay and Transwell assay showed that the migration and invasion abilities induced by TGF-β1 were significantly inhibited; Western blot and immunofluorescence experiments further confirmed that Abrine could up-regulate the expression of E-cadherin and down-regulate the expressions of N-cadherin, vimentin, Snail, and Slug, indicating that Abrine can effectively reverse the EMT process induced by TGF-β1, and may have the potential to inhibit the metastatic potential of tumor cells.

In the tumor microenvironment, the activity of Nrf2 can promote tumor chemotherapy resistance and stimulate tumor cell growth (37, 38), and Keap-1, as an inhibitor of Nrf2, regulates the stability and activity of Nrf2 (39, 40). In this study, the expression of Keap-1 was reduced: Keap-1 is the main negative regulator of Nrf2, responsible for targeting its target protein for proteasomal degradation. The reduction in Keap-1 levels directly stabilizes the Nrf2 protein, which is a key step in pathway activation. The overall increase in Nrf2 protein levels, together with the upregulation of expression of its classical target gene HO-1, Abrine treatment was associated with changes in protein expression consistent with modulation of the Keap-1/Nrf2/HO-1 axis, suggesting a potential involvement of this pathway. Furthermore, regarding the relationship between ERK inhibition and Nrf2 regulation, we further extended the discussion to elucidate the mechanistic link. Based on the available literature, the regulation of Nrf2 by ERK is thought to be an indirect rather than a direct phosphorylation event. Therefore, we hypothesized that when Abrine inhibits ERK activity, the inhibitory effect of ERK signaling on Nrf2 is diminished, thereby promoting Nrf2 stabilization and enhancing downstream HO-1 expression, ultimately potentially regulating tumor microenvironment and oxidative stress. (41, 42).

Currently, the clinical treatment of metastatic non-small cell lung cancer mainly relies on conventional chemotherapy, molecular targeted drugs (such as EGFR/ALK inhibitors) and immune checkpoint blockade therapy. Although these treatment strategies have improved the prognosis of patients to some extent, there are still some key limitations: chemotherapy often leads to severe systemic toxicity and multi-drug resistance; targeted therapy is limited by specific gene mutation patterns and inevitably leads to acquired resistance; immune therapy only provides durable benefits in a limited number of patients (43, 44).

In addition to the antioxidant activity and anti-proliferation properties of Abrine *in vitro* (12), compared with other natural alkaloids that promote tumor metastasis, most of the reported natural products only achieve the phenotypic regulation of EMT without clear direct targets. Abrine has a unique advantage: it may have a good interaction with ERK protein, which has been preliminarily verified by molecular docking, CETSA, DARTS, MST and other methods. Furthermore, this study also verified the *in vivo* efficacy of Abrine through animal experiments. A lung metastasis model in C57BL/6J mice was established by tail vein injection of B16-F10 melanoma cells. The results showed that the Abrine groups (20 mg/kg, 40 mg/kg) and the combination group with Sch772984 could significantly improve the trend of weight loss in mice, reduce the number of melanoma metastatic lesions in lung tissues, and decrease the wet lung weight and lung coefficient. HE staining results showed that Abrine had no obvious toxicity to the heart, liver, spleen, kidney, and brain tissues of mice. These results confirmed the anti-lung metastasis model effect and good safety of Abrine *in vivo*. In conclusion, Abrine is a promising candidate molecule for anti-metastasis.

However, this study did not directly detect intracellular ROS levels. Our mechanism study focused on ERK−mediated EMT regulation and confirmed that abrine inhibited TGF−β1−induced EMT through the ERK signaling pathway, supported by target binding, pathway analysis, EMT marker detection and migration assays. Although we also explored Nrf2−related pathways, without direct ROS data, we cannot definitively conclude that abrine reduces intracellular ROS or directly regulates oxidative stress. In addition, the functional dependence on ERK signaling was not fully verified due to the lack of ERK1 knockdown experiments. The upstream−downstream relationship between oxidative stress and EMT, as well as the crosstalk among ERK, oxidative stress and EMT, remain unclear. Therefore, future studies can perform Nrf2 nuclear translocation and ARE reporter assays to verify the Nrf2/Keap−1/HO−1 pathway, use fluorescent probes to quantitatively measure intracellular ROS, and conduct ERK1 knockdown experiments to confirm its role in the anti−EMT and anti−metastatic effects of abrine. These experiments will help clarify the regulatory network and upstream−downstream relationship among ERK, oxidative stress and EMT.

## Data Availability

The original contributions presented in the study are included in the article/supplementary material. Further inquiries can be directed to the corresponding authors.
